# An Unusual Case of the Successful Treatment of Carbapenem-resistant Ventriculitis with Intravenous Colistin

**DOI:** 10.7759/cureus.7120

**Published:** 2020-02-27

**Authors:** Azizi Abu Bakar, Kamalanathan Palaniandy, Farizal Fadzil, Ainul Syahril Jaafar, Sanmugarajah Paramasvaran

**Affiliations:** 1 Neurosurgery, Universiti Kebangsaan Malaysia Medical Center, Kuala Lumpur, MYS; 2 Neurosurgery, Fakulti Perubatan, Universiti Kebangsaan Malaysia, Kuala Lumpur, MYS; 3 Neurosurgery, Universiti Sains Malaysia, Kuala Lumpur, MYS

**Keywords:** colistin, ventriculitis, carbapenem, acinetobacter

## Abstract

Ventriculitis is a well-documented complication of ventriculostomy, which is difficult to treat and is associated with high rates of mortality. There is a growing trend of resistance among many organisms, such as Acinetobacter baumannii, in particular, to most antibiotics with the exception of colistin. It is thought that colistin has poor blood-brain barrier penetration; therefore, in cases of ventriculitis, it is preferentially administered via the intrathecal or intraventricular route. These routes, in turn, risk introducing infections, which may perpetuate the problem. We report a case of multidrug-resistant Acinetobacter baumannii ventriculitis, which was treated successfully with intravenous colistin monotherapy.

## Introduction

Ventriculitis is a well-established common complication of indwelling external ventriculostomy catheters, which has high morbidity and mortality. However, the use of external ventriculostomy catheters cannot be avoided in neurosurgical practice. Common indications are for temporary cerebrospinal fluid (CSF) diversion and intracranial pressure monitoring in trauma. Despite many advances being made to ventricular drainage in terms of materials and techniques, ventricular catheter-related infection remains to be a major source of morbidity among these patients. A local Malaysian study in 2009 reported a ventricular catheter infection rate of 32.2% [1]. The most common organism was Acinetobacter species at 39% of all infections followed by methicillin-resistant Staphylococcus aureus [1].

However, it has been noted there is an emerging trend of multidrug-resistant gram-negative ventriculitis, in particular, carbapenem-resistant Acinetobacter baumannii, which is often only sensitive to colistin [2]. Intravenous colistin, a polypeptide antimicrobial, is generally not encouraged due to its poor CSF penetration and relatively high toxicity; instead, intraventricular or intrathecal administration is encouraged [2-3]. There have been several cases of successful management with intraventricular colistin reported in the literature, each with its own unique features, which will be discussed. Through this report, we intend to add to this compendium of information.

## Case presentation

**Figure 1:**
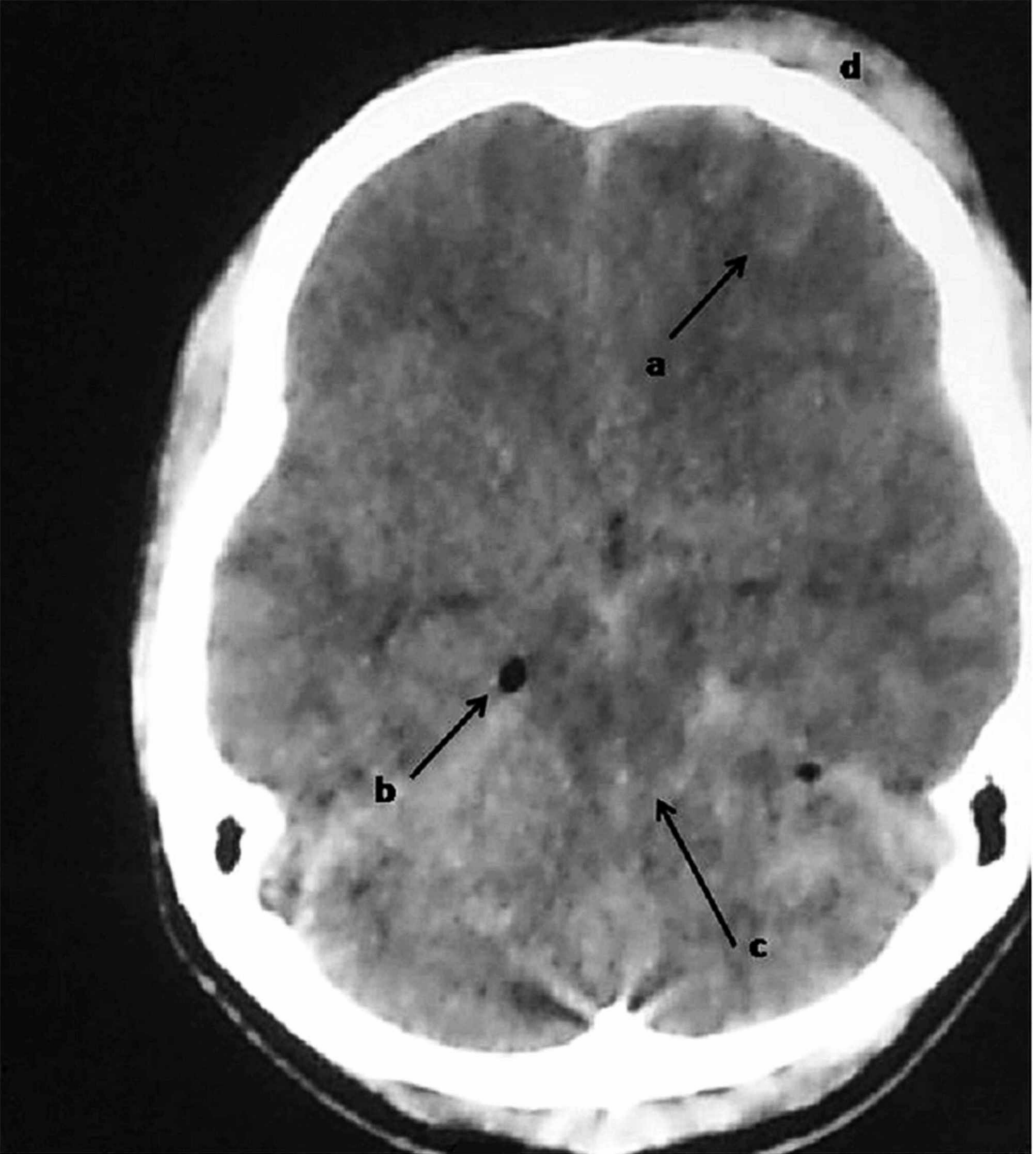
Computed tomography of the brain done at presentation (Day 1) The scan revealed: a - right frontal contusion; b - pneumocephalus; c - effacement of basal cisterns; d - right frontal subgaleal contusion

A 19-year-old male motorcyclist was involved in a road traffic accident. His Glasgow Coma Score (GCS) was 9/15 upon arrival at the hospital. He was intubated for airway protection. Computed tomography revealed the presence of right frontal contusion, pneumocephalus, and effacement of basal cisterns (Figure 1).

Emergent insertion of a right, frontal, external ventricular drainage catheter was performed. His intracranial pressure ranged between 2 mmHg and 16 mmHg with normal waveforms, was extubated at Day 3 post-trauma. It is the practice of our intensive care unit (ICU) to sample CSF in all patients with ventriculostomy catheters twice weekly, thus sampling was done at Days 3 and 6 post-ventriculostomy. The ventricular catheter was dislodged after sampling on Day 6, however, as the GCS was improving, we opted for clinical monitoring before deciding on the reinsertion of another external ventricular drain. Pyrexia was noted Day 2 onwards with neck stiffness, septic work was done and a third-generation cephalosporin was started on clinical and biochemical grounds. CSF culture and sensitivity result came back at Day 5; it was positive for carbapenem-resistant Acinetobacter baumannii, only sensitive to colistin.

We were advised to commence the patient on intrathecal colistin. However, his parents refused another surgery, therefore, high-dose colistin E (3 million units 8 hourly) was commenced and followed up closely clinically and biochemically via repeated lumbar punctures. Upon completion of two weeks of intravenous colistin and the normalization of biochemical parameters, as summarized in Table 1, the patient was discharged home with a Glasgow Outcome Score (GOS) of 5 [4].

**Table 1:** Summary of the cerebrospinal fluid culture as well as biochemical trend WCC - White Cell Count; CRP - C-Reactive Protein

Day	Culture	Antibiotic Sensitivity	Protein (g/dL)	Glucose (mmol/L)	WCC (x10^3^/µL)	CRP (mg/L)
3	Acinetobacter baumannii	Colistin	0.27	5.2	18.5	12.6
6	Acinetobacter baumannii	Colistin	0.67	2.4	12.6	7.4
10	No growth	-	0.88	1.5	8.0	3.0
13	No growth	-	0.22	2.5	7.5	1.7

## Discussion

Infection by carbapenem-resistant organisms, Acinetobacter baumannii, in particular, presents a challenge in its management. More so if it involves the central nervous system, which is a microenvironment unto itself due to the presence of the blood-brain barrier. A community-acquired Acinetobacter central nervous system infection is exceedingly rare, with a reported incidence rate of 0.2%, whereas in nosocomial infections, it is 3.6% [5]. A nosocomial Acinetobacter infection is almost exclusively related to neurosurgical procedures such as craniotomies, ventricular shunt procedures and catheter ventriculostomy [2,5-6]. The reported incidence of ventriculostomy catheter-related infection varies from as low as 1.6% to as high as 32.3% [1,7-11]. Whereas the reported mortality rate is as high as 72.7%, which was a retrospective data analysis of a heterogeneous group of patients with Acinetobacter baumannii central nervous system infection in a developing country [6].

Colistin, a polypeptide antimicrobial agent was first introduced in the 1940s [12]. Soon after the introduction, it started to fall out of favor, particularly due to the neuro, nephro, and myotoxicity reports that followed. This was accelerated by the introduction of newer potent antimicrobials with better safety profiles. At that time, in the golden era of antibiotics research and production, multidrug resistance was not given much attention [12]. The late 20th century saw the reemergence of interest in older, and thought to be more toxic, antimicrobials such as colistin, quinolone, and sulbactam, mainly owing to the surge of multidrug-resistant organisms.

In central nervous system infections requiring colistin, the preferred administration is via either the intraventricular or the intrathecal route. This is due to poor blood-brain barrier penetration, which was first reported in 1999 [13]. However, it is associated with a high incidence of chemical meningitis, which may be as high as 60% [14]. Cerebrospinal fluid penetration of colistin measured using high-performance liquid chromatography was only 5% [15]. The same study also suggested no difference in the cerebrospinal penetration of colistin in patients with meningeal inflammation and those without, suggesting the need for higher doses of colistin to be administered [15]. However, an earlier case report showed a cerebrospinal fluid penetration of 25% [16]. The pharmacokinetics of intraventricular/intrathecally administered colistin is not known; however, it requires the use of an indwelling catheter to deliver colistin, which itself is a risk factor for infection or colonization. 

In our review of literature, there have been a total of 12 cases of the successful treatment of carbapenem-resistant central nervous system infections with intravenous colistin [6,16-17]. Another case series reported five patients with carbapenem-resistant central nervous system infection, however, it did not distinguish between Acinetobacter baumannii and Pseudomonas aeruginosa [17].

A recent publication reported a similar case, where the patient was successfully treated with intraventricular colistin followed by intravenous administration. However, the total duration of antibiotics was for six weeks, inclusive of two weeks of intraventricular administration and was augmented with glycylcycline [18]. In our patient, the total duration of treatment was just two weeks, thus reducing the possibility of toxicity and resistance.

## Conclusions

In our patient, the infection was successfully treated with intravenous colistin monotherapy without the need for the increased dosage or use of adjuncts. The duration of antibiotic administration and hospital stay was also short at two weeks, with good recovery. Intravenous colistin monotherapy is an option in carbapenem-resistant Acinetobacter ventriculitis. However, this needs to be proven and reproduced in well-designed controlled studies before being the norm.
